# Fast and accurate variant identification tool for sequencing-based studies

**DOI:** 10.1186/s12915-024-01891-4

**Published:** 2024-04-22

**Authors:** Jeffry M. Gaston, Eric J. Alm, An-Ni Zhang

**Affiliations:** 1 Google, Cambridge, USA; 2 Department of Biological Engineering, Massachusetts Institute of Technology, Cambridge, USA; 3 Department of Biological Engineering, Center for Microbiome Informatics and Therapeutics, Massachusetts Institute of Technology, Cambridge, USA

**Keywords:** Genetic variants, Bioinformatics, Variant identification, Microbiome, SARS-CoV-2

## Abstract

**Background:**

Accurate identification of genetic variants, such as point mutations and insertions/deletions (indels), is crucial for various genetic studies into epidemic tracking, population genetics, and disease diagnosis. Genetic studies into microbiomes often require processing numerous sequencing datasets, necessitating variant identifiers with high speed, accuracy, and robustness.

**Results:**

We present QuickVariants, a bioinformatics tool that effectively summarizes variant information from read alignments and identifies variants. When tested on diverse bacterial sequencing data, QuickVariants demonstrates a ninefold higher median speed than bcftools, a widely used variant identifier, with higher accuracy in identifying both point mutations and indels. This accuracy extends to variant identification in virus samples, including SARS-CoV-2, particularly with significantly fewer false negative indels than bcftools. The high accuracy of QuickVariants is further demonstrated by its detection of a greater number of Omicron-specific indels (5 versus 0) and point mutations (61 versus 48–54) than bcftools in sewage metagenomes predominated by Omicron variants. Much of the reduced accuracy of bcftools was attributable to its misinterpretation of indels, often producing false negative indels and false positive point mutations at the same locations.

**Conclusions:**

We introduce QuickVariants, a fast, accurate, and robust bioinformatics tool designed for identifying genetic variants for microbial studies. QuickVariants is available at https://github.com/caozhichongchong/QuickVariants.

**Supplementary Information:**

The online version contains supplementary material available at 10.1186/s12915-024-01891-4.

## Background

Accurately identifying and characterizing genetic variants, such as point mutations and insertions/deletions (indels), holds immense significance in comprehensive genetic studies—tracking infectious diseases [1], studying evolutionary processes [2, 3], diagnosing genetic diseases [4], and developing targeted therapy [5]. Many microbiome studies rely on sequencing to conduct genetic studies, which often requires processing numerous sequencing datasets. This large volume of microbiome sequencing datasets calls for a variant identification tool that is fast, accurate, and robust.

The most widely used variant identification tool is bcftools [6] (with over one million installations between 2009 and 2021 [7]), especially nowadays in microbiome studies [8, 9]. Bcftools has been reported to outperform other tools in point mutation identification with the highest accuracy [10–12], such as GATK [13], VarScan2 [14], and InStrain [15]. However, the evaluation of variant identification tools on indels is relatively limited compared to point mutations; only a few studies investigated its performance in identifying indels, and those studies focused on specific types of sequencing data, such as RNA-seq [16]. In addition, bcftools requires significantly more time to run compared to tools that make use of multithreading [17, 18]. In particular, bcftools can use multiple threads for writing files, but does not take full advantage when processing files [17, 18].

In this study, we present QuickVariants, a fast, accurate, and robust tool for identifying genetic variants in sequencing-based microbiome studies. We compared the processing times of QuickVariants and bcftools on the same computer offering 10 GB RAM,15 threads, and 3.00 GHz CPU speed. It was observed that bcftools takes roughly 1 min (median) to process a 2–3 GB SAM file (containing 700 million to 1100 million bp). The runtime of bcftools exhibited a linear relationship with the size of the SAM file, necessitating 2 min for a 4 GB SAM file. Extrapolating to 1,000 SAM files of similar size, the processing time by bcftools would range between 16.7 and 33.3 h, a typical data volume in microbiome genetic research employing sequencing. In comparison, we estimate QuickVariants would complete the analysis within a timeframe of 1.6 to 2.9 h. Our tool is designed to process large-scale sequence data for microbial genetic research.

## Results

### QuickVariants algorithm and performance evaluation

QuickVariants is a computational tool designed to summarize variant information from read alignments without discarding (filtering out) reads. Its functionality is characterized by several distinct features:

- 1) Unlike bcftools, which uses complex models to produce metrics commonly employed in diploid organism studies like human genetics, such as GL, IMF, VOB, SGB, MQSB, and MQOF, QuickVariants emphasizes simplicity and generates metrics more relevant to microbial genetic studies. It outputs real counts, optimizing computational performance and simplifying user interpretation.
- 2) QuickVariants differentiates (via a separate output column) variants originating from the middle versus the end of a read alignment (within the first or last 10% of the read by default). This distinction, presented in a separate output column, is important as the middle section of a read can more confidently and accurately distinguish between an indel and point mutations than can the end of the read.
- 3) In the case of overlapping paired-end reads, QuickVariants consolidates the information, reporting a read depth of 1 rather than 2.
- 4) QuickVariants counts the total depth contributed by a specific position in a read (or read pair) as 1. Specifically, if a read aligns to N locations in the reference with optimal alignment, QuickVariants enumerates all alignments, reporting a read depth of 1/N for each.

To assess the effectiveness of QuickVariants and bcftools in detecting variants in sequence-based studies, we created a benchmark dataset using whole genome sequencing (WGS) of various bacterial species representing the human gut microbiome [19]. First, for each WGS sample, we aligned the WGS reads to a reference genome assembled from all reads, and corrected point mutations detected in the assembly by bcftools. This process resulted in reference genomes for which bcftools detected no variants when comparing the genomes to the reads (Methods).

Next, we artificially introduced in silico variants into the corrected assemblies (Additional file 1: Fig S1), including a series of simulations with varying densities of point mutations (ranging from 1e-6 to 5e-2) and 200 in silico insertions and deletions (indels) with various length (ranging from 2–17 bp) [20, 21]. These mutated genomes were then aligned to WGS reads using three distinct read aligners: Bowtie2 [22], Minimap2 [23], and BWA [24].

To evaluate the ability of QuickVariants and bcftools to detect the known in silico variants, we used the same read alignments as input for both tools. QuickVariants and bcftools summarized variant information from the read alignment results to identify variants. We compared the variants reported by both tools to the known in silico variants after applying identical variant filtering parameters. This comparison allowed us to evaluate the efficiency and accuracy of QuickVariants and bcftools in detecting variants in terms of their runtime, false positives, and false negatives.

### Speed of QuickVariants

**Fig. 1:**
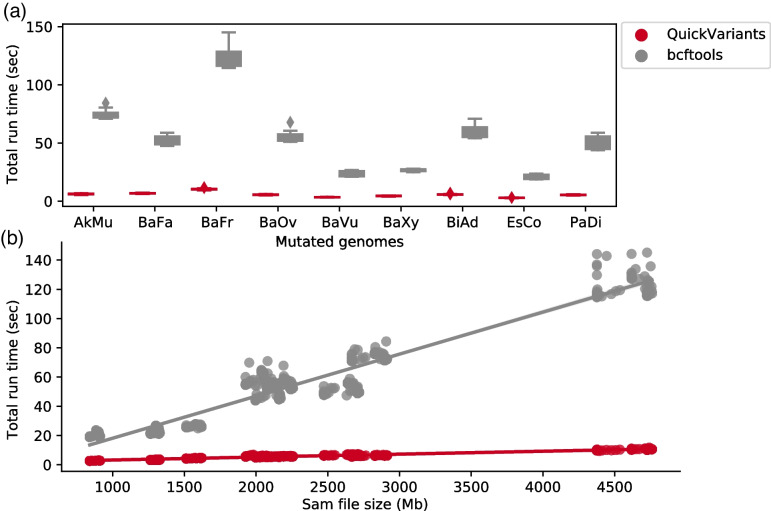
In a 15-thread evaluation on a computing node using 10 Gb RAM, QuickVariants exhibits a 9.2-fold acceleration in median runtime for generating variant reports compared to bcftools, using human gut microbiome WGS samples (raw read file size ranging from 0.7–3.6 Gb and genome size ranging from 2.2–7.1 Mbp). **a** Distribution of run time for QuickVariants and bcftools across various bacterial species. In box plots, error bars also indicate a 95% confidence interval (*n* = 57, 19 mutational densities and 3 aligners), and the central bars represent the median, excluding outliers. **b** Distribution of run time for QuickVariants and bcftools across different SAM file sizes. In point plots, error bars signify a 95% confidence interval, and the central points denote the mean

We found that QuickVariants’ 15-thread median runtime is 9.2-fold faster than the bcftools' median runtime for generating variant reports, e.g. Variant Call Format (VCF) files, from read alignments (Fig. 1a). On the same computing node with 10 Gb RAM and 15 threads, QuickVariants takes 2.9–10.5 s (95% confidence interval including outliers, with the median at 5.7 s) whereas bcftools takes 19.4–122.4 s (95% CI, median = 52.0 s) to process a bacterial genome (with genome size ranging from 2.2–7.1 Mbp and WGS sample size ranging from 0.7–3.6 Gb). Interestingly, our data reveal that bcftools runtime varies significantly across bacterial species, which is primarily attributed to changes in the size of the originating SAM files (Fig. 1b) with a slope of 0.029 s/Mb. In contrast, QuickVariants demonstrates greater resilience to SAM file size variations, displaying a slope of 0.002 s/Mb (Fig. 1b), which is 14.5-fold smaller than that of bcftools.

We further investigated why bcftools is slower compared to QuickVariants and we observed that the runtimes of bcftools were independent of the number of threads it was instructed to utilize. Specifically, the median runtime was 51.7 s (95% CI: 19.4–120.0 s) when instructed to use one thread (Additional file 1: Fig S2), and 52.0 s (95% CI: 19.4–122.4 s) when instructed to use 15 threads (Fig. 1). In comparison, the median single-threaded runtime of QuickVariants was only 22.3 s (95% CI: 11.5–41.0 s), which is 2.3-fold faster than the single-threaded runtime of bcftools (Additional file 1: Fig S2).

### Accuracy of QuickVariants to identify variants in human gut

**Fig. 2:**
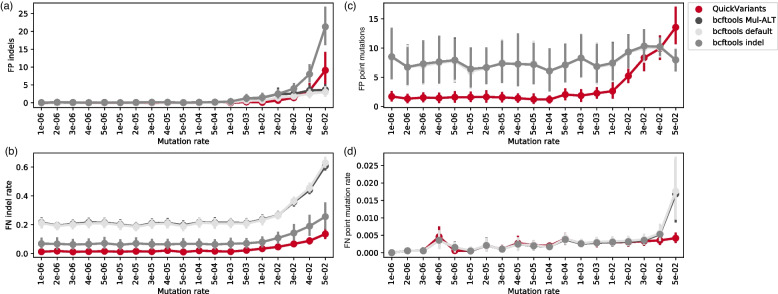
When tested against in silico variants in the human gut microbiome WGS samples, QuickVariants shows higher accuracy than bcftools by identifying (**a**) almost no false positive indels (0–2 FP indels, median = 0), (**b**) twofold to 16-fold fewer false negative indels (FN indels) (median), (**c**) 2.0-fold fewer median FP point mutations, and (**d**) 1.3-fold fewer median FN point mutations. **a**,**d** In point plots, error bars signify a 95% confidence interval, and the central points denote the mean values. Samples were labeled by the number of in silico point mutations inserted into the corrected assembly (*n* = 27, 9 WGS datasets and 3 aligners)

QuickVariants demonstrated a substantial advantage over bcftools in the detection of known in silico indels, achieving 16-fold fewer median false negatives in the human gut microbiome WGS samples we tested (ks test *p*-value = 5e-217, D = 0.89, two-way method). Specifically, QuickVariants shows a median FN indel rate of 1.5% (95% CI: 0.0%-10.5%), whereas bcftools (with settings for outputting multiple alternate alleles, Mul-ALT) yields a higher median FN rate of 23.5% (95% CI: 10.0%-54.5%) (Fig. 2d). Both QuickVariants and bcftools effectively avoid false positive (FP) indels, with median values of 0.0 FP indels (95% CI: 0.0–3.0 and 0.0–5.4, respectively) (Fig. 2a) in the human gut microbiome WGS samples (with genome size ranging from 2.2–7.1 Mbp and average read depth ranging from 56 to 232).

An additional test with bcftools using a new experimental indel identification model (–indels-2.0) [25] revealed that QuickVariants identified 2.3-fold fewer median FN rates (1.5%, 95% CI: 0.0%-10.5%) than bcftools (indel, 3.5%, 95% CI: 0.5%-28.5%). In the meanwhile, QuickVariants finds fewer FP indels (0.0–3.0 FP indels; 95% CI) compared to bcftools (indel), which displays 0.0–12.0 FP indels (95% CI). These observations suggest that indels in other microbial studies may have been largely underestimated as an evolutionary mechanism due to the tendency of bcftools to miss the majority of indels under most settings (further demonstrated by the analysis of SARS-CoV-2 longitudinal sewage metagenomes in the following sections).

In identifying point mutations, QuickVariants also shows higher accuracy in the human gut microbiome WGS samples than bcftools (Mul-ALT), by detecting 2.5-fold fewer median FPs compared to bcftools (Mul-ALT) (Figs. 2c and d). Our results demonstrate that QuickVariants reliably detects point mutations with high accuracy across an extensive range of mutation densities. From mutation densities as low as 1e-6 to as high as 3e-2, QuickVariants achieves a low number of FP point mutations (Fig. 2c) and a comparable FN rate (Fig. 2d) than bcftools (Mul-ALT). In particular, QuickVariants identifies 1.0 median FP point mutations (95% CI: 0.0–8.0), whereas bcftools (Mul-ALT) produces 4.0 median FP point mutations (95% CI: 0.0–28.1). Additionally, 53% (572 of 1,071) of the FP point mutations identified by QuickVariants were also detected by bcftools (Mul-ALT), as compared to 16% (572 of 3,484) in the reverse scenario. These findings highlight the versatility of QuickVariants in analyzing bacterial samples with varied evolutionary distances, from clonal strains separated by mutational densities of 1e-6 to distinct lineages diverging over mutational densities of 3e-2.

We tested bcftools using settings that are frequently employed in sequence-based evolutionary studies (Methods), which results in the output of multiple alternate alleles (Mul-ALT). We also assessed the performance of bcftools under default settings (bcftools default) and found that modifying the parameters to output multiple alternate alleles had a minimal impact on variant identification accuracy, with a 1.0-fold change in median values (Fig. 2).

Additionally, because bcftools is optimized for diploid organisms such as humans (with the default ploidy setting as 2), we investigated whether altering the ploidy setting could improve variant identification accuracy by bcftools. We found that altering the ploidy from haploid to diploid elevates the QUAL score (reflecting an increased confidence in variant calls), and affects other genotype likelihood calculations, while leaving read depth unchanged because it is a model-independent metric that simply counts the number of reads aligned to a specific genomic locus. Since most variants identified in a haploid setting already satisfy the QUAL threshold, this ploidy adjustment minimally affects the accuracy of variant identification. This indicates that further adjustments are needed for bcftools for optimal utility in microbial genetic research.

In our analysis, QuickVariants consistently demonstrates higher accuracy than bcftools in the identification of point mutations and indels irrespective of the short-read aligners employed, such as Bowtie2, Minimap2, and BWA (Additional file 1: Fig S3). More importantly, we noted that with bcftools, the results of different aligner tools manifest a significantly higher variance in accuracy, while QuickVariants provides more consistent outcomes. Regarding point mutation reporting via bcftools, Bowtie2 shows significantly more FPs than Minimap2 and BWA (ks test *p*-value = 1e-15, D = 0.56 for FPs generated by Bowtie2 versus the other two aligners, two-way method), whereas Minimap2 and BWA exhibit no significant differences (ks test *p*-value = 1.00, D = 0.04 for FPs; *p*-value = 1.00, D = 0.03 for FNs, two-way method). However, when employing QuickVariants for point mutation identification, Bowtie2 reveals no substantial differences compared to Minimap2 and BWA (ks test *p*-value = 0.14, D = 0.10 for FPs; *p*-value = 0.91, D = 0.05 for FNs, two-way method). Likewise, no significant differences were found between Minimap2 and BWA (ks test *p*-value = 0.12, D = 0.13 for FPs; *p*-value = 0.97, D = 0.05 for FNs, two-way method).

Regarding indels, the three aligners display markedly disparate accuracy levels. When analyzing alignments via QuickVariants or bcftools (indel), Bowtie2 yields significantly more FNs than Minimap2 and BWA (ks test *p*-value = 1e-15, D = 0.46; *p*-value = 1e-15, D = 0.93, respectively, two-way method), with no distinction shown between Minimap2 and BWA (ks test *p*-value = 0.97, D = 0.05; *p*-value = 1.00, D = 0.04, respectively, two-way method). These observations suggest that Minimap2 and BWA may be better suited as aligners for indel identification. Conversely, when analyzing alignments via bcftools (Mul-ALT and default), Bowtie2 manifests significantly fewer FNs compared to Minimap2 and BWA (ks test *p*-value = 1e-15, D = 0.67, two-way method), and Minimap2 reveals significantly fewer FNs than BWA (ks test *p*-value = 1e-5, D = 0.26, two-way method). This inconsistency in indel results across different settings of bcftools implies that bcftools (Mul-ALT and default) might have additional underlying factors leading to the misinterpretation of indels.

### Underlying factors reducing accuracy of bcftools in variant identification

**Fig. 3:**
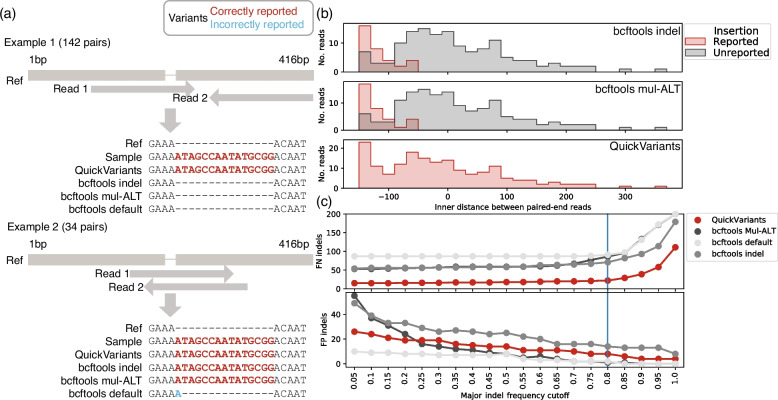
Bcftools excludes indels reported in input alignment results. **a** Illustration of two examples where bcftools excludes an insertion (Example 1) and reports a FP deletion (Example 2). A “-” represents a deletion. **b** The inner distance between paired-end reads influences the recognition of insertion by bcftools but not by QuickVariants. The inner distance of paired-end reads was computed as the distance between the alignment position of the end of read 1 and start of read 2. If two reads aligned to different contigs, the distance was computed as the closest distance between these positions in theory. **c** Indel identification in sample AkMu with a 4e-2 mutational density. The impact of the criterion for indel frequency on the accuracy of indel identification. The blue line represents the indel frequency threshold selected in this study

The reduced accuracy of bcftools in indel identification arises from inconsistencies between the variants in the input alignment data and the final output of bcftools. Specifically, bcftools ignores indels present in paired-end reads with minimal overlap, such as those with an inner distance exceeding -50 bp. For instance, Bowtie2 reported a total of 176 pairs of reads aligned to a reference with an insertion (in the reads) (Additional file 1: Fig S4a). These reads provide a comprehensive representation of the insertion, spanning various locations within the reads. The alignment data was processed by both QuickVariants and bcftools to produce variant reports. QuickVariants accurately identified insertions in all 176 reads. In contrast, bcftools (Mul-ALT and indel) recognized insertions in only 34 pairs of reads and excluded insertions in the remaining 142 pairs of reads (Fig. 3a, Example 1).

Interestingly, bcftools (Mul-ALT) appears to report insertions only when the paired-end reads are within an inner distance smaller than -50 bp, corresponding to an overlap of at least 50 bp in the case of 150 bp paired-end sequencing (Fig. 3b). It is noteworthy that overlapping reads are typically rare in paired-end sequencing, as library preparation often minimizes such overlaps. In addition, when using default settings, bcftools not only omitted the insertions but also incorrectly reported a 1-bp insertion in 34 instances (Fig. 3a, Example 2). This exclusion of indels by bcftools account for a significant proportion (18.3–45.1%) of the FN in silico indels (grey bars in Additional file 1: Fig S4b). Despite extensive exploration of bcftools settings, we failed to find a configuration that would accurately identify this insertion. This exclusion of indels occurred through the use of the command "bcftools call," which identifies variants from alignment results.

Additionally, exclusion of indels can also originate from the erroneous assignment of a period (“.”) to the ALT (alternative allele) field, inaccurately suggesting no variation at the position, despite the presence of an indel in the REF (reference) field. Such exclusion is executed through the "bcftools view" command, which is typically employed subsequent to "bcftools call" for variant filtration based on parameters like quality score (QUAL) and raw read depth (DP). In our examined sample, this issue resulted in the omission of 37.2–49.5% of indels under default settings (represented by the blue bars in Additional file 1: Fig S4b).

The exclusion of indels in bcftools leads to reduced depth support for indels, usually resulting in a decrease in indel frequency, and consequently causing the indel to be disqualified. Specifically, under the Mul-ALT settings, indels in bcftools were primarily screened out due to low quality, with indel frequency playing a considerable role. This is responsible for 53.5% and 79.7% of the FN in silico indels (bcftools Mul-ALT and indel, red bars in Additional file 1: Fig S4b). In exploring the dynamics of FN and FP in indel identification, we fine-tuned the indel frequency criterion. We found that a 0.8 indel frequency threshold effectively maintains a low FN rate while simultaneously minimizing FP occurrences, establishing it as an optimal cutoff (Fig. 3c). Regardless of adjustment to the indel frequency criterion, QuickVariants consistently demonstrates a reduction in the number of FN indels compared to bcftools, showing a 3.6- to 5.8-fold reduction at a 5% indel frequency, and a 1.6- to 1.8-fold reduction at a 100% indel frequency. Across various bcftools configurations, the indel setting consistently yielded the lowest FN rate, independent of the indel frequency criterion. In terms of FP indels, QuickVariants displays a 1.8- to 2.0-fold lower rate compared to bcftools (indel). These results underscore that QuickVariants is more precise in indel identification than bcftools, irrespective of the indel frequency criterion.

In contrast, QuickVariants successfully avoided 71.4%-87.9% of the FN in silico indels reported by bcftools. By employing read alignments only from the middle regions of reads (e.g. 80% of read length), QuickVariants enhances indel identification accuracy by circumventing problems that occur when the surrounding context is insufficient to distinguish indels from a series of point mutations. This method increased the accuracy of QuickVariants by 23.7%-41.9% in reducing FN in silico indels reported by bcftools (Additional file 1: Fig S4c).

The misinterpretation of indels by bcftools also led to most of the FP point mutations (10 of 10 cases we manually checked). In our analysis, reads containing a deletion aligned to the reference genome were either directly excluded or reported with zero read depth by bcftools. The exclusion of the deletion underreported the major variant at the reference positions. On the same reference positions, minor variants like point mutations were properly reported (supported by read depth). The absence of the major variant inflated the variant frequency of these minor variants, thereby facilitating FP point mutations by reaching the variant frequency threshold.

**Fig. 4:**
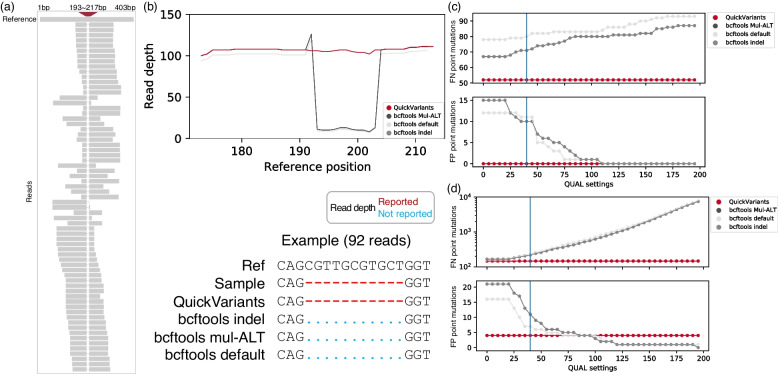
Bcftools reported zero read depth for the deletion region in the reference genome, leading to the underrepresentation of the major variant (the deletion). This absence of the major variant subsequently enables the occurrence of FP point mutations (minor variants) by meeting the variant frequency threshold. **a** Sequence alignment reveals a deletion in 92 reads (partially displayed) in reference positions 193–217 bp when aligned to the reference genome. **b** Illustration of an example where bcftools reported zero read depth of this deletion (the major variant) in 92 reads. A “.” represents that the read depth of the deletion region was not reported. A “-” represents that the read depth of the deletion region was correctly reported. **c**,**d** The impact of the QUAL criterion on the accuracy of point mutation identification by bcftools. The QUAL reported by bcftools indicates the Phred-scaled quality score for a variant, reflecting the confidence in the existence of the variant at that position. The blue line represents the QUAL threshold for bcftools selected in this study. QuickVariants neither compute QUAL nor utilize QUAL for variant filtering. **c** Effect of the QUAL criterion on the accuracy of point mutation identification by bcftools in sample BaOv with a 1e-06 mutational density. **d** Effect of the QUAL criterion on the accuracy of point mutation identification by bcftools in sample AkMu with a 4e-2 mutational density

For example, Bowtie2 identified a deletion supported by 92 reads (Fig. 4a) and QuickVariants accurately reported this deletion in all these reads (Fig. 4b). In contrast, bcftools did not recognize this deletion in 72 reads, reporting zero read depth for reference positions 193–217 bp. Additionally, bcftools reported the deletion in 20 reads, yet indicated zero depth for the reference positions covering the deletion region. Consequently, the major variant (the deletion) was underrepresented with zero read depth reported by bcftools.

In the meanwhile, 10 reads ending at the deletion region were interpreted as point mutations (minor variants) by Bowtie2. Both QuickVariants and bcftools (Mul-ALT and indel) reported these point mutations in 10 reads. QuickVariants, noting the support from 92 reads for the deletion (the major variant), classified these point mutations as low quality. However, bcftools, ignoring the major variant support, reported FP point mutations with a variant frequency of 100% (10 of 10 reads). Correctly reporting the deletion with an alignment depth of 20 reads would have prevented bcftools from producing these false positive point mutations by yielding a disqualified variant frequency of 33.3%.

Our investigation of point mutation filtering parameters, including the QUAL, demonstrates that altering this criterion (from the default of 40) did not improve bcftools' accuracy in point mutation identification. Bcftools reports a QUAL which indicates the confidence of a specific variant. QuickVariants does not compute or use QUAL for variant filtering. In two different samples, we adjusted the QUAL settings to study its effect to the FN and FP point mutations identified by bcftools. In Sample 1, QUAL adjustments did not reduce FP and FN point mutations in bcftools to a level comparable to that of QuickVariants (Fig. 4c). In Sample 2, although adjusting the QUAL criterion effectively reduced FP point mutations, it led to a significant rise in FN point mutations (Fig. 4d). Specifically, the number of FPs decreased from 16–21 to 0–1 when the QUAL threshold was altered from 0 to 195, whereas FNs rose from 167–169 to 7,465–7,862. QuickVariants, in comparison, reported only 4 FPs and 147 FNs. At a QUAL threshold of approximately 85, bcftools yielded 4 FPs but had 3.2- to 3.6-fold more FNs (471–534 point mutations) than QuickVariants.

### Coverage of QuickVariants to identify SARS-CoV-2 variants

To evaluate the robustness of QuickVariants to handle microbial species beyond bacteria, we evaluated the ability of QuickVariants and bcftools to identify artificially in silico variants within the SARS-CoV-2 Wuhan-1 sample. This earliest-isolated strain serves as a critical baseline for comparative genomic analyses of SARS-CoV-2^24^. First, we analyzed the genetic differences between all published genomes of SARS-CoV-2 strains from the GISAID database [13] (08/2023) to the original Wuhan-1 genome. We found that most dissimilarities are confined to a maximum of 120 point mutations and 10 indels. Then, we systematically introduced a range of point mutations (from 5 to 300) and 20 indels into the Wuhan-1 assembly to simulate SARS-CoV-2 evolution. Then we aligned the Wuhan-1 WGS sample to the mutated Wuhan-1 assembly to identify those known variants. As bcftools reported no variants between the Wuhan-1 WGS sample and the original Wuhan-1 assembly, no assembly correction was required.

**Fig. 5:**
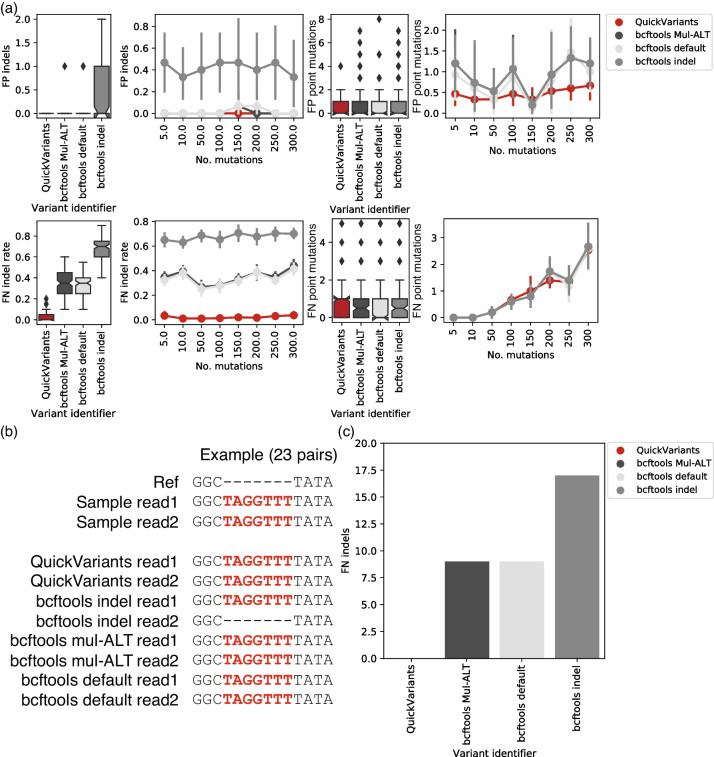
When tested against in silico SARS-CoV-2 variants, QuickVariants demonstrates higher accuracy compared to bcftools by identifying (**a**) almost no FP indels (0.0–1.0 for 95% CI, median = 0.0), 4.3-fold fewer median FN indels, almost no false positive (FP) point mutations (0.0–1.0 for 95% CI, median = 0.0), and no false negative (FN) point mutations (0.0–3.0 for 95% CI, median = 1.0). **a** Distribution of FP indels and FN indel rates identified by QuickVariants and bcftools. Variance in FP indels and FN indel rates identified by QuickVariants and bcftools at different mutation frequencies, with datasets labeled by the number of in silico point mutations inserted into the corrected assembly. Distribution of FP point mutations and FN point mutation rates identified by QuickVariants and bcftools. Variance in FP point mutations and FN point mutation rates identified by QuickVariants and bcftools at different mutation frequencies, with datasets labeled by the number of in silico point mutations inserted into the corrected assembly. In both point and bar plots, error bars signify a 95% confidence interval (*n* = 30 for point plots and *n* = 240 for bar plots, 8 mutational densities, 10 replicates, and 3 aligners), and the central points denote the mean. For box plots, error bars also indicate a 95% confidence interval, and the central bars represent the median, excluding outliers. **b**,**c** Indel identification in sample with 200 in silico point mutations and 20 in silico indels. **b** Illustration of three examples where bcftools (indel) excludes the insertion for read 2. A “-” represents a deletion. **c** FN indels identified by QuickVariants and bcftools. Both QuickVariants and bcftools identified 0 FP indels in this sample

We found that QuickVariants demonstrates higher accuracy than bcftools (Mul-ALT and indel) by nearly eliminating all FN indels for SARS-CoV-2 sequencing data (ks test *p*-value = 3e-63, D = 0.97, and *p*-value = 2e-71, D = 1.0, two-way method, respectively) (Fig. 5a). Specifically, QuickVariants demonstrates a substantially lower median FN indel rate of 0.0% (95% CI: 0.0%-10.0%), compared to the 35.0% (95% CI: 15.0%-50.0%) exhibited by bcftools (Mul-ALT and default). In the meanwhile, both QuickVariants and bcftools effectively minimize the identification of FP indels with a median of 0.0 (95% CI: 0.0–0.0).

The indel setting in bcftools significantly elevates both the FN indels (95% CI: 50.0%-85.0%, median = 70.0%; ks test *p*-value = 1e-44, D = 0.85, two-way method) and FP indels (95% CI: 0.00–1.05, median = 0.00; ks test *p*-value = 3e-07, D = 0.36, two-way method) compared to that of bcftools (Mul-ALT and default) for SARS-CoV-2 sequencing data (Fig. 5a). We observed inconsistent indel frequency reporting between bcftools (Mul-ALT) and bcftools (indel). For instance, in a sample where Minimap2 identified 98 read pairs with insertions aligned to a reference, QuickVariants accurately identified insertions in all 98 pairs. In contrast, bcftools (Mul-ALT) detected insertions in only 42 pairs of reads and excluded insertions in the remaining 56 pairs. Interestingly, bcftools (indel) excluded insertions for the second read (read2) in 23 pairs (Fig. 5b), leading to a reduced indel frequency of (19*2 + 23)/(42*2) = 0.72. Under a 0.8 indel frequency threshold, this indel was deemed as low quality and consequently screened out in the final result, categorizing it as a FN indel. This led to a higher number of 17 FN indels identified by bcftools (indel) in this sample, compared to 9 FN indels reported by bcftools (Mul-ALT and default). QuickVariants, however, completely avoided the FN indels reported by bcftools (Fig. 5c).

The findings demonstrate that QuickVariants is an effective and reliable tool for the detection of SARS-CoV-2 variants, exhibiting exceptional sensitivity and accuracy compared to bcftools.

### Performance of QuickVariants to identify SARS-CoV-2 variants in longitudinal metagenomes

To evaluate the performance of QuickVariants in identifying variants in real samples, we downloaded longitudinal metagenomes from sewage in Wisconsin, USA, collected in January 2022 and March 2023 [26]. The SARS-CoV-2 Delta and Omicron BA.1 lineages were previously reported as the dominant variants of concern in January 2022, whereas the Omicron BA.5, BQ.1, and XBB.1.5 lineages were identified as the dominant variants of concern in March 2023 [26]. Thus, we summarized variants unique to Omicron BA.1 lineage compared to Delta and B.1.1 lineages (the closest non-Omicron ancestor) [27] and classified these variants as the “old Omicron variants” (65 point mutations and 53 indels). Similarly, we summarized variants unique to Omicron BA.5, BQ.1, or XBB.1.5 lineages compared to Omicron BA.1 lineage [27] and classified these variants as the “new Omicron variants” (56 point mutations and 12 indels). We expect a significant enrichment of Omicron variants at both time points, with the "new Omicron variants" more likely to be detected at the second time point (March 2023).

We found that QuickVariants identified 3–6 additional old Omicron point mutations (out of 37) in January 2022 and 4–7 additional new Omicron point mutations (out of 24) in March 2023, compared to bcftools under various settings (Additional file 1: Fig S5a). The point mutations detected by QuickVariants were significantly enriched in variants unique to Omicron (*p* < 1E-104, Fisher's exact tests compared to any random point mutations), indicating that these mutations are more likely true positives rather than random sequencing errors. By tightening the filtering threshold for allele frequency from 20 to 50% to detect point mutations present in a larger proportion of the human population, QuickVariants identified 1–4 additional old Omicron point mutations (out of 34) in January 2022 and 2–4 additional new Omicron point mutations (out of 20) in March 2023, compared to bcftools under different settings (Additional file 1: Fig S5b). Meanwhile, QuickVariants reported 0–1 fewer non-Omicron variants than bcftools. These results suggest QuickVariants is more accurate than the bcftools by minimizing both false positives (missed Omicron variants) and false negatives (non-Omicron variants caused by sequencing errors).

As to indels, only QuickVariants detected Omicron-specific indels, identifying 2 out of 10 total indels in January 2022 as Omicron-specific and 3 out of 4 new indels in March 2023 as Omicron-specific (*p* = 7E-6 and *p* = 2E-10, respectively, Fisher's exact test compared to indels at random positions). In contrast, bcftools (under various settings) identified one non-Omicron indel in January 2022 and no new indels in March 2023. These observations further support the possibility that indels may have been significantly underestimated as an evolutionary mechanism, due to the tendency of bcftools to miss the majority of indels under most settings.

## Discussion

We found that QuickVariants and bcftools reported no false positives in sequencing data simulated with sequencing errors. We evaluated QuickVariants using simulated illumine WGS data, with sequencing error rates varying from 0.1 to 10 times the original error rate, and 20X read depth [28] (Additional file 1: Fig S6a). Similarly, we assessed the tools on simulated illumine metagenomic data, with the original sequencing error rate and 20X read depth (Additional file 1: Fig S6b). After filtering the variants using the same criteria for WGS and metagenomic studies (e.g. 20% allele frequency and 4 reads, which equals 20% of 20X read depth, details in Methods), we observed that both QuickVariants and bcftools (under various settings) reported no false positives for point mutations and indels. Given that genetic studies typically apply their own variant filtering criteria to the reported results, QuickVariants was designed to present the original results without pre-filtering the variants with undefined criteria to avoid confusion. More importantly, the default filtering criteria of bcftools, which are not clearly explained, do not effectively reduce false positives compared to QuickVariants. Instead, they may lead to an increase in false positive point mutations (Fig. 2c), primarily due to incorrectly interpretation of indels.

However, QuickVariants does not help distinguish between low-confidence variants from sequencing errors, such as variants at low allele frequencies (< 20%) or in genomic regions with low read depth. We lowered the variant filter criteria from 20% allele frequency and 4 reads to 5% allele frequency and 1 read for both simulated WGS and metagenomic datasets. This adjustment did not result in any false positive point mutations and indels in WGS datasets. Nevertheless, for metagenomic datasets, QuickVariants identified false positive point mutations and indels, which decreased with stricter filtering criteria (Additional file 1: Fig S6b): from an average of 453,439 point mutations and 21 indels at 1 read and 5% allele frequency to an average of 794 point mutations and 2.7 indels at 2 reads and 10% allele frequency, further reducing to an average of 4 point mutations and 0 indels at 3 reads and 15% allele frequency, and ultimately to 0 point mutations and 0 indels at 4 reads and 20% allele frequency.

To further evaluate the sensitivity of QuickVariants to sequencing errors in high-depth metagenomic datasets, we simulated an illumine metagenomic dataset with the original sequencing error rate and a 100X read depth. Applying the same filtering criteria as before (e.g., 20% allele frequency and 20 reads, which equals 20% of 100X read depth), we observed no false positives for point mutations and indels detected by both QuickVariants and bcftools across various settings (Additional file 1: Fig S6c). However, lowering the filtering threshold to 5% allele frequency and 5 reads resulted in QuickVariants reporting an average of 31.3 point mutations and 4.6 indels as false positives. Thus, QuickVariants does not help differentiate variants with low allele frequencies (< 20%) and variants on genomic regions with low read depth (e.g. 1 variant read at a position with ≤ 5 total reads, leading to an allele frequency of ≥ 20%) from sequencing errors. Regardless of the tool reporting them, variants identified with low allele frequencies should be carefully verified via alternative sequencing or experimental methods, as such low-frequency variants identified in next-generation sequencing data are often subsequently disconfirmed by Sanger sequencing [29, 30].

In addition, we observed that the majority of false positives identified by QuickVariants originated from SAM files produced by minimap2, representing 11 out of 17 scenarios where QuickVariants reported false positives. This observation suggests that QuickVariants demonstrates greater robustness to sequencing errors when analyzing outputs from bowtie2 and bwa (Additional file 1: Fig S6).

## Conclusions

In this study, we introduce QuickVariants, a fast, accurate, and robust bioinformatics tool designed for identifying genetic variants for microbial studies, including both point mutations and indels. When tested using human gut microbiome WGS samples, QuickVariants processes data nine times faster than bcftools when utilizing 15 threads. Furthermore, QuickVariants consistently exhibits lower rates of false positives and negatives compared to bcftools under equivalent variant filtering conditions. This consistent accuracy is observed across multiple alignment tools and is notably evident in data aligned by Bowtie2. Importantly, QuickVariants also shows applicability in detecting variants in viral sequences with high accuracy, including those of SARS-CoV-2.

We observed that bcftools ("bcftools call" command) frequently excludes indels in minimally overlapping paired-end reads. This exclusion led to the non-reporting of 18.3% to 45.1% of indels and the disqualification of 53.5% to 79.7% of indels (for having low frequencies), resulting in false negatives in indel identification. Additionally, the default setting of the "bcftools view" command excluded 37.2% to 49.5% of qualified indels by erroneously assigning a period (“.”) to the ALT (alternative allele) field. The exclusion of indels by bcftools also leads to an underreporting of the major variant, which in turn elevates the frequency of minor variants, contributing to false positives in point mutation identification in 10 out of 10 cases we examined. Furthermore, QuickVariants enhances indel detection accuracy by utilizing read alignments from the middle region of reads. This approach reduces the incidence of bcftools-reported FNs in indel identification by 71.4% to 87.9%.

QuickVariants offers speed, accuracy, and robustness for diverse microbial sequencing samples. It is applicable in various microbial genetic research areas including epidemic tracking, population genetics, disease diagnosis, and targeted therapy.

## Methods

### Benchmarking datasets of the human gut microbiome for performance evaluation

To evaluate the performance of QuickVariants and bcftools, we downloaded nine whole genome sequencing (WGS) datasets (short reads and reference genomes assembled from the short reads), covering diverse human gut microbiome species^18^, including SRX5976902 *Akkermansia muciniphila* (*AkMu*), SRX597774 *Bacteroides* faecis (*BaFa*), SRX5976649 *Bacteroides fragilis* (*BaFr*), SRX5976729 *Bacteroides ovatus* (*BaOv*), SRX6045315 *Bacteroides vulgatus* (*BaVu*), SRX6044844 *Bacteroides xylanisolvens* (*BaXy*), SRX6044813 *Bifidobacterium adolescentis* (*BiAd*), SRX5991169 *Escherichia coli* (*EsCo*), and SRX5992782 *Parabacteroides distasonis* (*PaDi*). Most WGS datasets have a beyond adequate sequencing depth of more than 1 Gb of sequence data (1.08–3.60 Gb).

To remove potential false positive point mutations caused by assembly errors, we first corrected point mutations reported by bcftools in the original assemblies. To do that, the WGS short reads were aligned to the original assemblies by Bowtie2, Minimap2, and BWA and identified point mutations by bcftools. Point mutations were filtered by the same criteria that we used to identify in silico point mutations, and all genomic positions with qualified point mutations or under supported alignments (low major variant frequency of 80%) were corrected by converting to ambiguous base pair “N”, to generate corrected assemblies. As a result, bcftools reports no variants when aligning the WGS reads to the corrected assemblies from read alignments generated from any aligner.

Then in silico variants were randomly introduced into each corrected assembly (Additional file 1: Fig S1), including a series of mutational densities of 1e-6, 2e-6, 3e-6, 4e-6, 5e-6, 1e-5, 2e-5, 3e-5, 4e-5, 5e-5, 1e-4, 5e-4, 1e-3, 5e-3, 1e-2, 2e-2, 3e-2, 4e-2, and 5e-2. Particularly, we found that all aligners (Bowtie2, Minimap2, and BWA) show a dramatic drop in alignment quality (< 50% reads aligned) for genomes with more than 5e-2 mutational density. In addition, in silico 200 indels were randomly introduced into each mutated genome. The length of indels was randomly sampled from 2–20 bp because the majority (90%) of indels in prokaryotic genomes were reported to have a small size (< = 15 bp) [20, 21], and the frequency of indels drops with indel size following a power law [31]. QuickVariants and bcftools detected comparable numbers of indels in the original assemblies: 0.0–21.1 (95% CI, median = 1.0) and 0.0–42.0 (95% CI, median = 0.0), respectively (ks test *p*-value = 0.6, D = 0.16, two-way method). Thus, we excluded indels found in the original assemblies from those identified in mutated genomes.

The WGS short reads were aligned to the series of genomes with known mutations by Bowtie2, Minimap2, and BWA to generate read alignment results – SAM files. The SAM files were then converted to variant reports by QuickVariants and bcftools. The performance was evaluated in terms of runtime (elapsed to execute), the number of false positives (FPs), and the number of false negatives (FNs). For a fair comparison of their runtimes, QuickVariants and bcftools were run on the same dataset sequentially (one QuickVariants job followed by one bcftools job) on the same computer offering 10 Gb of RAM,15 threads, and 3.00 GHz CPU speed.

### Benchmarking SARS-Cov-2 dataset for performance evaluation

To evaluate the ability of QuickVariants to handle other microbial species such as viruses, we downloaded the SARS-Cov-2 WGS datasets of the Wuhan-Hu-1 lineage [32] from NCBI (NC_045512.2 and SRR10971381, 20 Gb in total). According to our investigation into the genetic differences between the original Wuhan-1 assembly and the most recent variants of SARS-CoV-2 strains (08/2023), the majority of dissimilarities are confined to a maximum of 120 point mutations and 10 indel mutations (ranging from 3 to 26 bp) [13]. Thus, we randomly introduced a series of in silico point mutations (5, 10, 50, 100, 150, 200, 250, and 300) and 20 in silico indels of varying lengths (ranging from 2 to 20 bp) into the Wuhan-1 assembly. To comprehensively evaluate the ability of QuickVariants to identify variants, we generated 5 randomly mutated genomes for each mutational density.

### Short read alignment and bcftools

For short read alignments, we used Bowtie2 [22] version 2.5.1 (default settings), BWA [24] v0.7.17-r1188 (default settings), and Minimap2 [23] v2.26-r1175 (*-ax sr -N 10 -p 0.9*). To generate variant reports, e.g. vcf files, from the SAM outputs of those aligners, we used the latest Samtools [33] v1.18 (*samtools sort -n -O SAM* before QuickVariants and *samtools sort -O SAM* before bcftools) and the latest Bcftools [34] v1.18 (Mul-ALT settings *bcftools mpileup -A | bcftools call -A -M*) [35, 36], Bcftools [34] v1.18 (indel identification model settings *–indels-2.0*) and Bcftools [34] v1.18 with default settings. The ploidy in bcftools call was set as haploid for microbiome species (*–ploidy 1*). The Mul-ALT settings of bcftools only function when the -A option is set for both “bcftools mpileup” and “bcftools call”. We also evaluated the Bcftools v1.18 (increasing max depth from default 250 reads to 3000 reads by *bcftools mpileup -d3000*; setting ploidy as diploid by removing *–ploidy 1*) and Bcftools v1.9 for sample AkMu with a 4e-2 mutational density. For point mutation identification, we ran the command of *bcftools view -H -v snps -i 'QUAL* >  = *20 & MIN(DP)* >  = *3'*. Likewise, for indel identification, we ran the command of *bcftools view -H -v indels -i 'QUAL* >  = *20 & MIN(DP)* >  = *3'*.

### Point mutation and indel filtering

We applied the same criteria, based on the protocol of previous studies [35–38], to filter for point mutations and indels using the variant reports files generated by QuickVariants and bcftools. Briefly, low-quality point mutations were screened out if they had 1) low mapping quality (< 40 Qual, bcftools only); 2) low depth support (< 3 reads); 3) low point mutation frequency (< 90% for bacterial WGS and 70% for SARS-CoV2 WGS); or 4) presence within the last 10 bp of a contig. Similarly, low quality indels were screened out with the criteria of 1) low mapping quality (< 20 Qual, bcftools only); 2) low depth support (< 6 reads); or 3) low indel frequency (< 80%).

Note that for computing total depth of raw reads mapped to a genomic position, we account for the depth of both point mutations and indels. Similar to most sequence alignment approaches, we set a higher penalty for gap opening than gap extension [22, 39] when filtering for qualified indels. Thus, for QuickVariants, we asked for >  = 80% variant frequency for the start of an indel, and >  = 70% variant frequency for the extension of an indel. In addition, QuickVariants does not output alignment quality scores (Qual) as bcftools. Instead, QuickVariants distinguishes between the middle and the end of a read that supports the reference variant for each reference position. Since the middle of a read contains more evidence from the neighborhood to support an indel than the end of a read, we focused on the middle of reads when filtering for indels.

### Simulation data with sequencing errors

To evaluate the ability of QuickVariants and bcftools to handle sequencing errors, we simulated illumine Hiseq 2500 and NextSeq 500 v2 WGS datasets (*art_illumina -ss HS25 -p -l 150* and *-ss NS50 -p -l 75*) with sequencing error rates varying from 0.1 to 10 times the original error rate *(-qs -10*, *-qs 1*, and -*qs 10*), and 20X read depth or coverage (*-f 20*) [28]. The WGS simulation datasets were generated from a reference genome of *B. fragilis* NCTC 9343 (NCBI accession GCF_000025985.1) by ART v2.5.8 [40]. Similarly, we simulated illumine Hiseq 2500 metagenomic datasets (*art_illumina -ss HS25 -p -l 150*) with the original sequencing error rate (*-qs 1*): one with a 20X read depth (*-f 20*) and another with a 100X read depth (*-f 100*). The metagenomic simulation datasets were generated from nine human gut microbiome reference genomes. For metagenomic datasets, low-quality point mutations were screened out if they had 1) low mapping quality (< 40 Qual, bcftools only); 2) low point mutation frequency (< 20%); 3) low depth support (< 4 reads for the 20X-depth dataset and < 20 reads for the 100X-depth dataset, equivalent to 20% out of 20 total reads and 20% out of 100 total reads); or 4) presence within the last 10 bp of a contig. Likewise, low quality indels were screened out with the criteria of 1) low mapping quality (< 20 Qual, bcftools only); 2) low indel frequency (< 20%); or 3) low depth support (< 4 reads for the 20X-depth dataset and < 20 reads for the 100X-depth dataset, equivalent to 20% out of 20 total reads and 20% out of 100 total reads).

### Collecting SARS-Cov-2 longitudinal metagenomic dataset for performance evaluation

To evaluate the performance of QuickVariants in identifying variants in real samples, we downloaded longitudinal metagenomes from Wisconsin, USA sewage, collected in January 2022 (NCBI Accession SRR21019653, SRR21019687) and March 2023 (NCBI Accession SRR23934917) [26]. The “old Omicron variants” were identified as variants unique to Omicron BA.1 lineage (represented by strain BA.2) compared to Delta (represented by strain B.1.617.2) and B.1.1 (the closest non-Omicron ancestor, represented by strain B.1.1.529) [27]. Similarly, the “new Omicron variants” were identified as variants unique to Omicron BA.5 (represented by strain BA.5.2.1), BQ.1 (represented by strain BQ.1.1.1), or XBB.1.5 (represented by strain XBB.1.5) compared to Omicron BA.1 (represented by strain BA.2) [27]. We summarized 65 old Omicron point mutations, 56 new Omicron point mutations, 53 old Omicron indels, and 12 new Omicron indels (Additional file 2).

Sewage metagenomes were aligned to the SARS-Cov-2 reference genome (NC_045512.2) to generate vcf files using the same method described before. Due to the high depth of these metagenomes, the maximum depth parameter of bcftools was increased from 250 (default) to 300,000 reads (via *bcftools mpileup -d300000*). Point mutations with low quality were excluded based on the following criteria: 1) mapping quality < 40 Qual (bcftools only); 2) point mutation frequency < 20%; 3) depth support < 20 reads (equivalent to 20% of 100 total reads); or 4) presence within the last 10 bp of a contig. Likewise, low-quality indels were screened out if they exhibited 1) mapping quality < 20 Qual (bcftools only); 2) indel frequency < 20%; or 3) depth support < 20 reads (equivalent to 20% of 100 total reads). We selected a 20% allele frequency as the cutoff because nearly all variants identified in next-generation sequencing data and subsequently disconfirmed by Sanger sequencing were found to have an allele frequency of less than 20% [29].

### Supplementary Information

**Additional file 1:** **Figure S1**-Evaluation of effectiveness and accuracy of QuickVariants and bcftools for variant detection. **Figure S2**-Distribution of single-threaded run time for QuickVariants and bcftools across various bacterial species. **Figure S3**-When tested on alignment results from Bowtie2, Minimap2, and BWA, QuickVariants demonstrates higher accuracy than bcftools in identifying point mutations and indels. **Figure S4**-Bcftools excludes indels reported in input alignment results. **Figure S5**-QuickVariants identified more Omicron-specific variants (point mutations) than bcftools in longitudinal sewage metagenomes. **Figure S6**-False positive point mutations and indels reported by QuickVariants and bcftools in sequencing data simulated with sequencing errors.

**Additional file 2:** Classification of Omicron point mutations and indels detected in January 2022 and March 2023 from Wisconsin, USA sewage.

## Data Availability

All data generated or analysed during this study are included in this published article, its supplementary information files and publicly available repositories. All code is publicly available on https://github.com/caozhichongchong/QuickVariants or 10.5281/zenodo.10982271 [41]. Code was tested on openjdk 11.0.20, python v3.10.9, and jupyter notebook v5.7.1. All sequencing datasets used in this study are publicly available on 10.6084/m9.figshare.25437217 [42], including. 1) the benchmark datasets (raw reads, genome assemblies, genomes with known in silico mutations, and the mapping data of inserted point mutations and indels); 2) WGS and metagenomic datasets simulated with sequencing errors; 3) longitudinal SARS-Cov-2 sewage metagenomes and the reference genome 4) the SAM files and variant reports that support the examples shown in Figs. 3– 5.

## Abbreviations

ALT: Alternative allele
DP: Read depth
FP: False positive
FN: False negative
Indels: Insertions/deletions
Mul-ALT: Multiple alternate alleles
QUAL: Quality score
REF: Reference
WGS: Whole genome sequencing
