# Supplementary material for: Fast and accurate variant identification tool for sequencing-based studies
Source: BMC Biol. 2024 Apr 22;22:90. doi: 10.1186/s12915-024-01891-4 (PMC11034086; doi:10.1186/s12915-024-01891-4)
Supplement: Supplementary file 1 — Additional file 1: Figure S1-Evaluation of effectiveness and accuracy of QuickVariants and bcftools for variant detection. Figure S2-Distribution of single-threaded run time for QuickVariants and bcftools across various bacterial species. Figure S3-When tested on alignment results from Bowtie2, Minimap2, and BWA, QuickVariants demonstrates higher accuracy than bcftools in identifying point mutations and indels. Figure S4-Bcftools excludes indels reported in input alignment results. Figure S5-QuickVariants identified more Omicron-specific variants (point mutations) than bcftools in longitudinal sewage metagenomes. Figure S6-False positive point mutations and indels reported by QuickVariants and bcftools in sequencing data simulated with sequencing errors. [file 12915_2024_1891_MOESM1_ESM.docx]

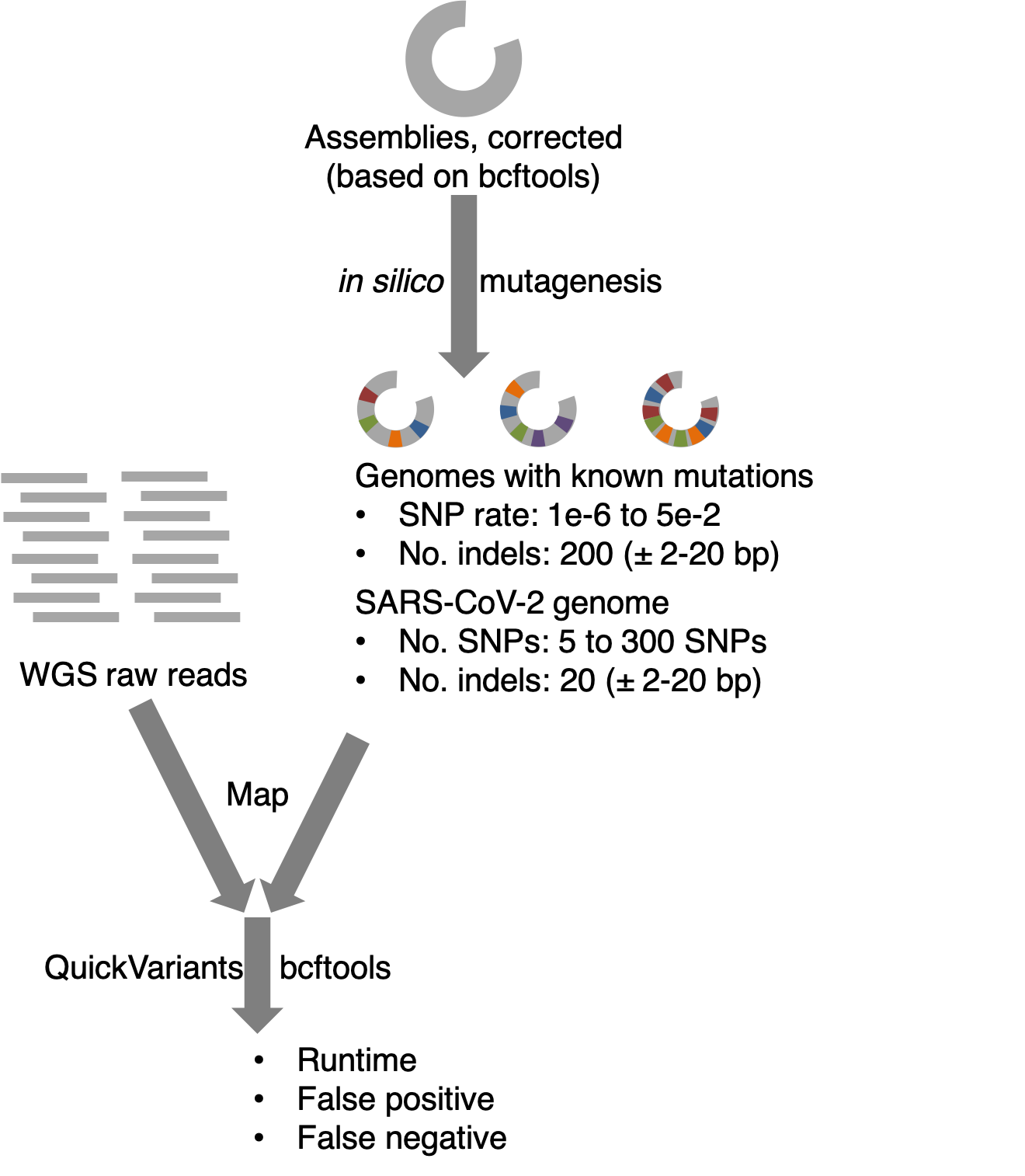


Figure S1. Evaluation of effectiveness and accuracy of QuickVariants and bcftools for variant detection using short-read samples obtained from whole genome sequencing (WGS) and corresponding assemblies of various bacterial species representing the human gut microbiome.


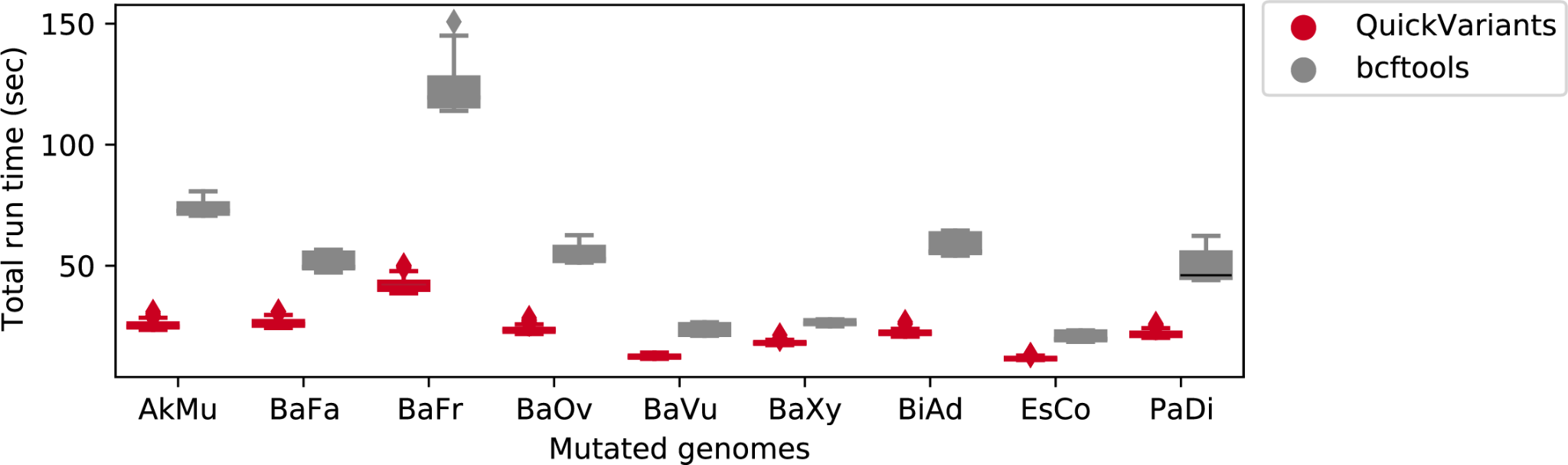


Figure S2. Distribution of single-threaded run time for QuickVariants and bcftools across various bacterial species. Single-threaded QuickVariants exhibits a 2.3-fold acceleration in median runtime for generating variant reports compared to bcftools, using the same human gut microbiome WGS samples as in Figure 1 on a computing node offering with 10Gb RAM. In box plots, error bars also indicate a 95% confidence interval (n = 57, 19 mutational densities and 3 aligners), and the central bars represent the median, excluding outliers.


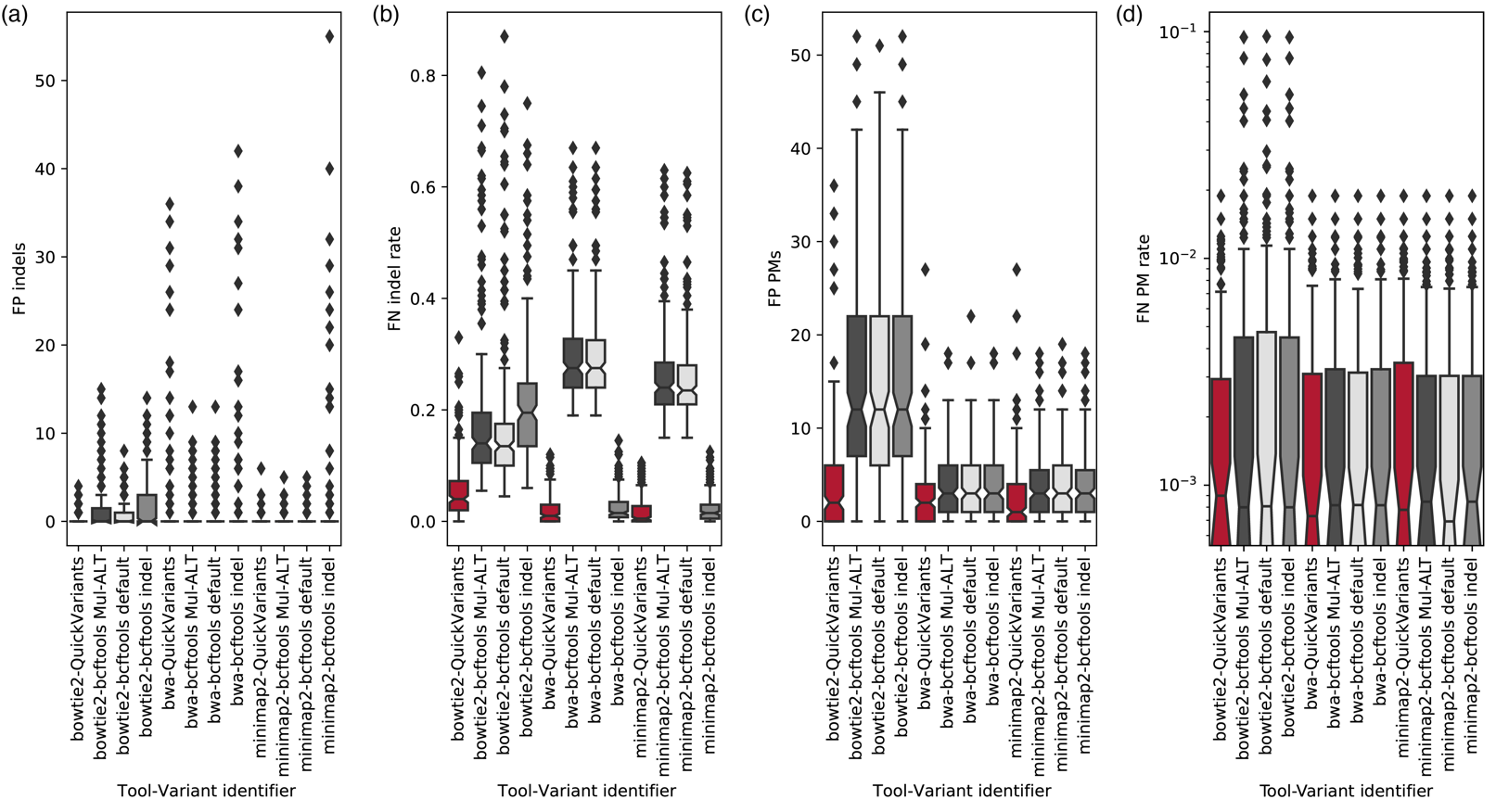
Figure S3. When tested on alignment results from Bowtie2, Minimap2, and BWA, QuickVariants demonstrates higher accuracy than bcftools by identifying (a) false positive (FP) point mutations, (b) false negative (FN) point mutations, (c) false positive indels, and (d) false negative indels when identifying *in silico* variants in the human gut microbiome. In both point and bar plots, error bars signify a 95% confidence interval, and the central points denote the mean. For box plots, error bars also indicate a 95% confidence interval (n = 171, 9 WGS datasets and 19 mutational densities), and the central bars represent the median, excluding outliers.


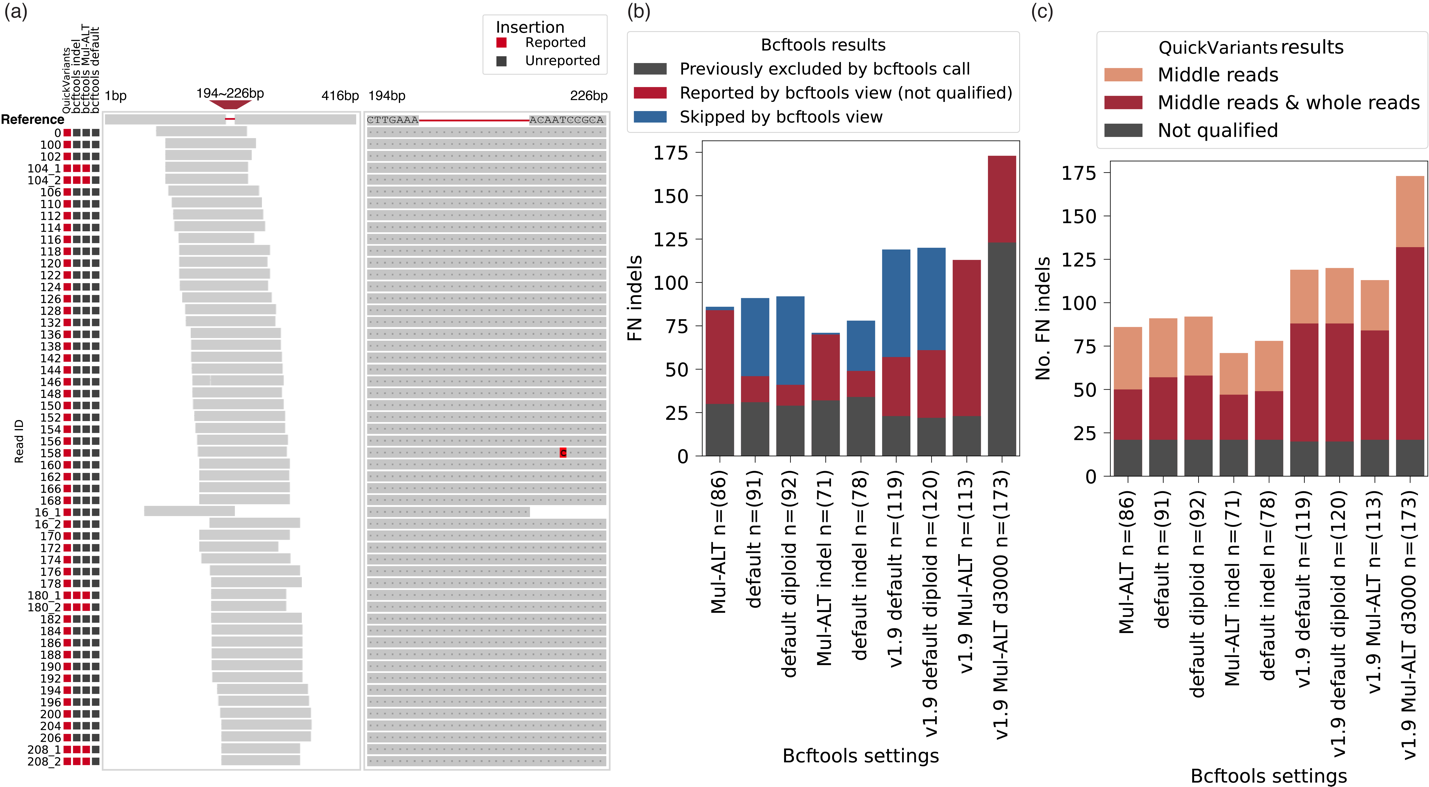
Figure S4. Bcftools excludes indels reported in input alignment results. (a) Comparison of results between QuickVariants, bcftools (indel), bcftools (Mul-ALT), and bcftools (default), in recognizing an insertion within 176 pairs of reads (partially displayed) aligned to a reference. (b-c) Indel identification in sample AkMu with a 4e-2 mutational density. (b) FN *in silico* indels reported by bcftools under various settings due to exclusion by commands “bcftools call” or “bcftools view”. (c) The ability of QuickVariants to identify indels that were missed by bcftools (FN *in silico* indels), with filtering based on read alignments from the middle of reads (“middle reads”), or the entire reads (“whole read”).

**
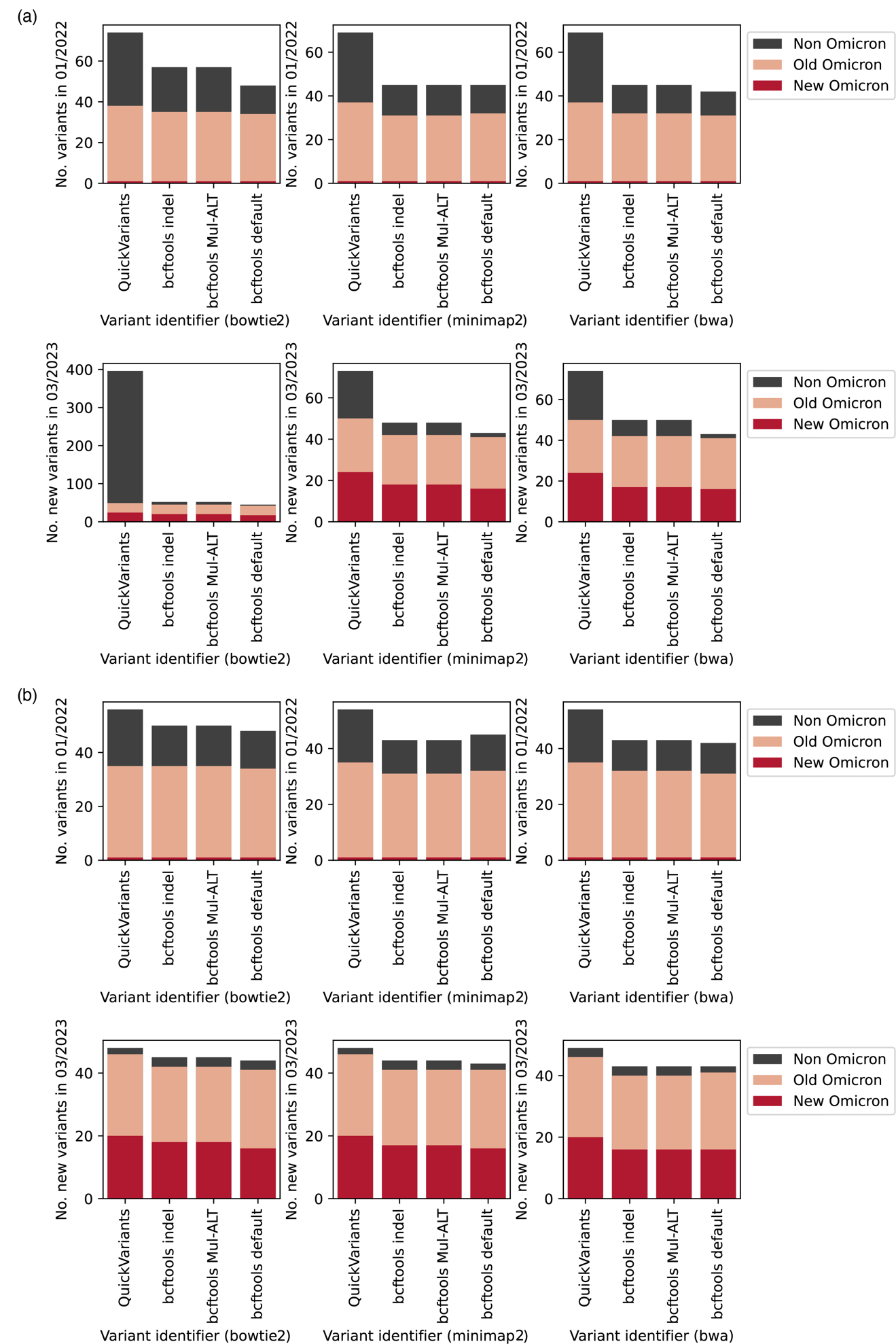
**

Figure S5. QuickVariants identified more Omicron-specific variants (point mutations) than bcftools in longitudinal sewage metagenomes from Wisconsin, USA, collected in January 2022 (01/2022) and March 2023 (03/2023). The SARS-CoV-2 Delta and Omicron BA.1 lineages were the dominant variants of concern in January 2022, whereas the Omicron BA.5, BQ.1, and XBB.1.5 lineages were the dominant variants of concern in March 2023. Variants unique to Omicron BA.1 lineage compared to Delta and B.1.1 lineages were classified as “old Omicron variants”. Variants unique to Omicron BA.5, BQ.1, or XBB.1.5 c lineages compared to Omicron BA.1 lineage were classified as the “new Omicron variants”. Variants that do not belong to either “old Omicron variants” or “new Omicron variants” were classified as “non Omicron variants”. The comparison utilized alignment results from Bowtie2, Minimap2, and BWA. (a) The number of point mutations detected in 01/2022 (upper panel) and point mutations newly detected in 03/2023 (lower panel, compared to 01/2022) by QuickVariants and bcftools under different settings. Point mutations were filtered with the criteria of 20 read depth and 20% allele frequency. (b) The number of point mutations detected in 01/2022 (upper panel) and point mutations newly detected in 03/2023 (lower panel, compared to 01/2022) by QuickVariants and bcftools under different settings. Point mutations were filtered with the criteria of 20 read depth and 50% allele frequency.

**
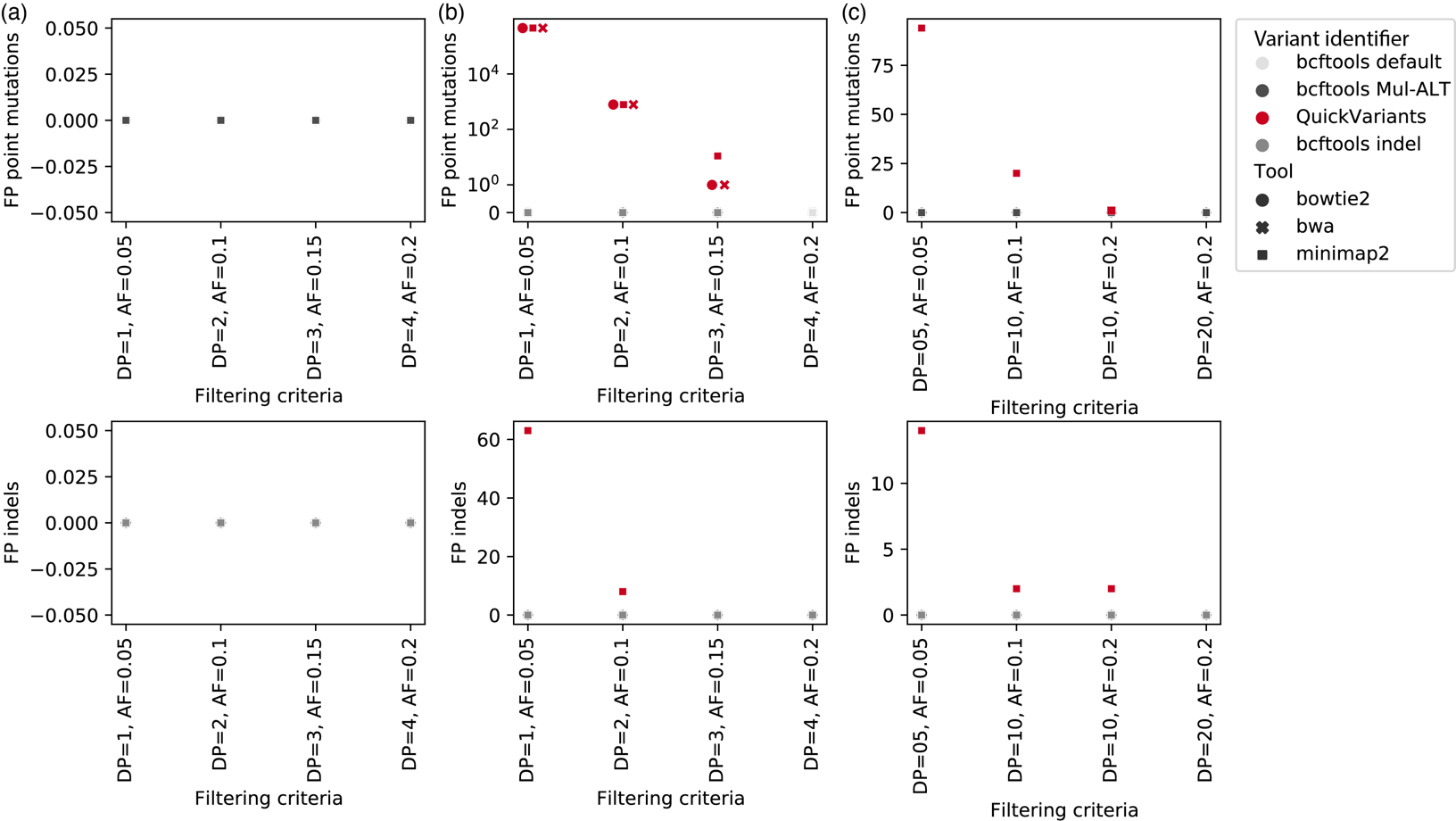
**

Figure S6. False positive (FP) point mutations and indels reported by QuickVariants and bcftools in sequencing data simulated with sequencing errors. Variants were filtered by different criteria, including read depth (DP) and allele frequency (AF). (a) The number of false positive point mutations (upper panel) and indels (lower panel) identified by QuickVariants and bcftools in WGS data simulated with sequencing error rates varying from 0.1 to 10 times the original error rate, and 20X read depth. Variants generated from different Illumina sequencing platforms and different datasets simulated with various sequencing errors were pooled together. (b) The number of FP point mutations (upper panel) and indels (lower panel) identified by QuickVariants and bcftools in metagenomic data with the original sequencing error rate and 20X read depth. (c) The number of FP point mutations (upper panel) and indels (lower panel) identified by QuickVariants and bcftools in metagenomic data with the original sequencing error rate and 100X read depth.
